# Randomized clinical trial in cancer patients shows immune metabolic effects exerted by formulated bioactive phenolic diterpenes with potential clinical benefits

**DOI:** 10.3389/fimmu.2025.1519978

**Published:** 2025-02-17

**Authors:** Marta Gómez de Cedrón, Juan Moreno-Rubio, Victor de la O Pascual, Beatriz Alvarez, Marta Villarino, María Sereno, César Gómez-Raposo, Silvia Roa, Miriam López Gómez, María Merino-Salvador, Ana Jiménez-Gordo, Sandra Falagán, Cristina Aguayo, Francisco Zambrana, Beatriz Tabarés, Beatriz Garrido, Silvia Cruz-Gil, Cristina M. Fernández Díaz, Lara P. Fernández, Susana Molina, María Carmen Crespo, Youness Ouahid, Juan José Montoya, Ricardo Ramos Ruíz, Guillermo Reglero, Ana Ramírez de Molina, Enrique Casado

**Affiliations:** ^1^ Molecular Oncology Group, IMDEA Food, CEI UAM+CSIC, Madrid, Spain; ^2^ Medical Oncology Department, Infanta Sofia University Hospital-Henares University Hospital-Foundation for Biomedical Research and Innovation (FIIB HUIS HHEN), Madrid, Spain; ^3^ Clinical Oncology Group, IMDEA Food, CEI UAM+CSIC, Madrid, Spain; ^4^ Precision Nutrition and Cardiometabolic Health, IMDEA Food, CEI UAM+CSIC, Madrid, Spain; ^5^ Faculty of Health Sciences, International University of La Rioja (UNIR), Logroño, Spain; ^6^ Centro Nacional de Investigaciones Cardiovasculares CarlosIII (CNIC Carlos III), Madrid, Spain; ^7^ MiRNAX Biosens Research & Development Unit (MBR&DU), Madrid, Spain; ^8^ Faculty of Medicine, School of Sport Medicine, Universidad Complutense de Madrid, Madrid, Spain; ^9^ Institute of Food Science Research CIAL CSIC-UAM, Madrid, Spain; ^10^ Production and Development of Foods for Health, IMDEA Food, CEI UAM+CSIC, Madrid, Spain

**Keywords:** immunology, precision nutrition, clinical trial, cancer, bioactive compounds

## Abstract

**Background:**

Nutrients, including bioactive natural compounds, have been demonstrated to affect key metabolic processes implicated in tumor growth and progression, both in preclinical and clinical trials. Although the application of precision nutrition as a complementary approach to improve cancer treatments is still incipient in clinical practice, the development of powerful “omics” techniques has opened new possibilities for delivering nutritional advice to cancer patients. Precision nutrition may contribute to improving the plasticity and function of antitumor immune responses.

**Objectives:**

Herein, we present the results of a randomized, prospective, longitudinal, double-blind, and parallel clinical trial (NCT05080920) in cancer patients to explore the immune-metabolic effects of a bioactive formula based on diterpenic phenols from rosemary, formulated with bioactive alkylglycerols (Lipchronic^©^ WO/2017/187000). The trial involved cancer patients, including those with lung cancer (LC), colorectal cancer (CRC), and breast cancer (BC), undergoing chemotherapy, targeted biological therapy, and/or immunotherapy. The main readouts of the study were the analysis of Lip on systemic inflammation, hemogram profile, anthropometry, lipid and glucose profiles, and tolerability. Additionally, a deep immune phenotyping of peripheral blood mononuclear cells (PBMCs) was performed to identify the functional effects of Lip on key mediators of the immune system.

**Results:**

Lip was well tolerated. The lung cancer subgroup of patients showed a reduction in biomarkers of systemic inflammation, including the neutrophil-to-lymphocyte ratio (NLR). Furthermore, modulation of key players in the immune system associated with the experimental treatment Lip compared to the control placebo (Pla) treatment was revealed, with particularities among the distinct subgroups of patients. Our results encourage further research to apply molecular nutrition-based strategies as a complementary tool in the clinical management of cancer patients, particularly in the current era of novel immunotherapies.

**Clinical trial registration:**

ClinicalTrials.gov, identifier NCT05080920

## Introduction

Nutrients affect fundamental metabolic cellular processes, and some diet-derived ingredients, including bioactive compounds and extracts from natural sources, have been demonstrated to inhibit tumor growth in both preclinical and clinical trials (1–6). Powerful “omics” techniques -genomic, transcriptomic, proteomic, metabolomic, lipidomic, and metagenomic analyses- have opened new avenues in nutritional sciences for the delivery of nutritional advice through precision nutrition (7).

Previously, we described rosemary (Rosmarinus officinalis L.) supercritical extract (SFRE), approved for human consumption and rich in phenolic diterpenes, as a potential antitumor agent in colorectal cancer (CRC), breast cancer (BC), and non-small cell lung cancer (NSCLC). This is due to its effects on the inhibition of lipid metabolism and the reduction of molecular targets implicated in systemic inflammation (8–11). Moreover, SFRE synergized with chemotherapeutic and immune checkpoint inhibitors *in vitro*, inhibiting the proliferation of CRC, BC, and NSCLC cells (1, 12, 13).

Importantly, a clinical trial in healthy volunteers with SFRE formulated with bioactive alkylglycerols to improve the bioavailability of the SFRE bioactive compounds, Lipchronic^©^ (WO/2017/187000), confirmed the safety and tolerability of the intervention. Lipid metabolic-associated targets implicated in inflammation were reduced, and modulation of key players of the immune system with potential benefits in cancer were observed (14).

Tumor immunology describes the dynamic interaction between the immune system and tumor cells, and understanding these interactions is crucial for developing complementary therapies in cancer treatment. Additionally, the tumor immune microenvironment (TME) is highly variable among patients and depends not only on intrinsic cancer-associated alterations but also on additional factors such as nutritional status, which is affected by lifestyle characteristics (i.e., diet, exercise) and influences the response to clinical treatments (7, 15). Thus, malnutrition and/or visceral obesity may promote immune system dysfunction, triggering systemic inflammation frequently associated with a variety of adverse events, such as delayed recovery and even mortality in cancer (16).

Moreover, the role of cancer-associated inflammation in prognosis is well established, and a variety of biochemical and hematological parameters, such as the number of inflammatory cells (neutrophils, lymphocytes, monocytes) alone or in the form of their ratio (neutrophil-to-lymphocyte ratio, NLR), including platelet counts, are considered inflammation-associated biomarkers in some illnesses (17–19), and predictors of several malignancies, including cancer (20), metabolic disorders (21), and cardio-cerebral events (22).

Recently, an advanced machine learning model from the cross-sectional analysis of transcriptomic and proteomic data of nearly 7,500 oncologic patients in three major cohorts -lung cancer (LC), colorectal cancer (CRC), and breast cancer (BC)- and the reported molecular mechanism of action of extracts from rosemary found a high correlation between cancer-triggering points of inflammation and immune system constriction with the molecular effects of diterpenic phenols from rosemary (23).

We conducted a randomized, prospective, longitudinal, double-blind, and parallel pilot clinical trial (NCT05080920) in collaboration with the Medical Oncology Department of Infanta Sofia Hospital (Madrid, Spain). The trial involved cancer patients undergoing chemotherapy, targeted biological therapy, and/or immunotherapy. The objective was to evaluate the effects of Lipchronic^®^ (Lip) on systemic inflammation, hemogram profile, anthropometry, lipid and glucose profiles, and tolerability. Additionally, a deep immune phenotyping of peripheral blood mononuclear cells (PBMCs) was performed to identify the functional effects of Lipchronic^®^ on key mediators of the immune system.

## Materials and methods

### Study design

This clinical trial was conducted by the Medical Oncology Department of Hospital Universitario Infanta Sofia (Madrid, Spain) in collaboration with the IMDEA-Food Institute. A total of 108 patients undergoing cancer treatments -including chemotherapy, immunotherapy, and/or targeted therapies- were recruited to evaluate the functional and immune metabolic effects of Lipchronic^©^ (WO/2017/187000), a bioactive formula based on phenolic diterpenes from rosemary supercritical extract (SFRE) formulated with bioactive alkylglycerols (WO/2017/187000), previously evaluated in healthy volunteers (14).

Initially, as the study was undertaken during the COVID-19 pandemic, one of the primary endpoints was to evaluate the effects of Lipchronic^©^ in the prevention of infections in cancer patients undergoing treatment. However, due to the effective hygiene measures and vaccines during the COVID-19 pandemic, patient infections were significantly reduced. Consequently, the study was closed early after 108 patients had been randomized, and this objective was eliminated from the study.

Main objectives of this clinical trial were to evaluate the effects of Lipchronic^©^ on systemic inflammation, parameters related to quality of life (Short Form Questionnaire 36, SF36), symptoms, anthropometry, biochemistry, and tolerability. Additionally, a deep immune phenotyping of peripheral blood mononuclear cells (PBMCs) was performed to identify the functional effects on key mediators of the immune system.

The randomization procedure was provided by the Biostatistics Unit of the IMDEA Food Institute. It was carried out using a randomization table generated by Statistic Software R version 2.15 (www.r-project.org, University of Auckland, Auckland, New Zealand). Clinical and pathological data were collected from medical reports.

The setup of this clinical trial was a randomized, prospective, longitudinal, double-blind, and parallel pilot study with two study arms: Lipchronic^©^ (Lip) and control placebo capsules (Pla). The complete description of the composition of Lip and Pla capsules was previously described (14). Briefly, rosemary supercritical extract (SFRE) contained 12-16% phenolic diterpenes (carnosic acid and carnosol, calculated as carnosic acid, with >10% carnosic acid), total volatile flavor compounds <4%, water <1%, residual content of ethanol <2%, and cuticular waxes, as provided by Flavex (Rosemary Extract 25, Type No. 027.020, sourced from Flavex Naturextrakte GmbH, Rehlingen-Siersburg, Germany, extracted using supercritical CO_2_ extraction methods).

SFRE is a dietary supplement in concentrations authorized by EFSA, so no toxicities were expected. According to the NOAEL (No Adverse Effect Level) for SFRE -180 to 400 mg of extract/kg body weight/day-, the dose in this study was calculated considering the lower end of the range (180 mg/kg/day) and dividing by a safety factor of 200, based on a calculated weight of 50 kg. This results in a dose of 45 mg/extract/day, whose bioavailability will be enhanced by the inclusion of the lipid vehicle (alkylglycerols) in the formulation, thus being a dose within the range authorized by EFSA for food use. Patients were instructed to consume one capsule per day. A consumption questionnaire was used to monitor adherence to the treatments, with the consumption of capsules or non-compliance recorded daily. Additionally, patients were asked to deliver unconsumed capsules at each visit to verify intake.

The clinical trial was approved by the Ethics Committee for Clinical Research of La Paz University Hospital (Ref. HULP 5617) and was carried out in accordance with the Code of Ethics of the World Medical Association (Declaration of Helsinki). Written informed consent was obtained from all subjects prior to starting the trial.

### Inclusion and exclusion criteria

Inclusion Criteria: Patients of both sexes aged between 18 and 85 years with a diagnosis of solid tumor undergoing active antitumor treatment (including chemotherapy, targeted therapies, immunotherapy, and hormone therapy), whether for disseminated disease or perioperative management of localized disease. No history of having had COVID-19 (confirmed or probable: positive PCR test/criteria based on clinical presentation with IgM+/pneumonia with lymphopenia and elevated d-dimer levels and negative procalcitonin). Adequate understanding of the clinical study. Willingness to voluntarily participate in the study and provide written informed consent.

Exclusion Criteria: Subjects who refuse to consume the study product or have a fish allergy. Subjects who consume vitamin or antioxidant supplements and are unwilling to stop taking them one week before and during the treatment. Dysphagia of any origin that, in the investigator’s judgment, could hinder treatment ingestion. Bilirubin levels >1.5 times the upper limit of normal (ULN). ALT and AST levels >2.5 times ULN. Serum creatinine >1.5 times ULN and/or creatinine clearance <50 ml/min. Severe organ dysfunction based on symptoms, signs, laboratory studies, or rapid disease progression (life expectancy less than 4 months). Uncontrolled hypertension (blood pressure readings of 150/95 mmHg despite optimal treatment). NYHA class II or higher heart failure. Before or after 6 months of coronary bypass surgery. Atrial fibrillation with a heart rate >100 bpm. Unstable ischemic heart disease. Severe pre-existing conditions that, in the investigator’s judgment, would preclude inclusion in the study (e.g., history of major duodenal resection affecting the absorption of the lipid carrier of the study product). Cholelithiasis, biliary obstruction, and cholangitis. Currently receiving investigational treatment in a clinical trial or study deemed incompatible with this trial. Immunodeficiencies and inflammatory diseases such as HIV infection, collagen disorders, and inflammatory bowel disease. Dementia and severe psychiatric disorders. Pregnancy, lactation, or plans for either during the study. For women of childbearing age, a negative pregnancy test was required, and appropriate contraceptive measures (pre-existing requirements for oncological treatment).

### Data collection from patients

The SF36 (Short Form 36) questionnaire during the study allowed the collection of quality information related to Health-Related-Quality-of-Life of patients. More specifically, the validated version for Spanish population of the SF36 Health Survey was used (24). SF36 integrates eight concepts of health: physical functioning (PF), bodily pain (BP), role limitations due to physical health problems (RP), role limitations due to personal or emotional problems (RE), general mental health (MH), social functioning (SF), energy/fatigue or vitality (VIT), and general health perceptions (GH). Emotional well-being and vitality have been used interchangeably with general mental health and energy/fatigue, respectively. The items contributing to a scale were scored so that a higher score represents better health, and they were averaged together to create the scale score. For each parameter, scores were coded, summed, and transformed to a scale from 0 (the worst possible condition) to 100 (the best possible condition). SF36 questionnaire was surveyed at baseline visit 1 (V1) (treatment onset as reference category), at V4-nine weeks of intervention, at V6-sixteen weeks of intervention, and at V7-twelve weeks post-intervention.

In addition, a total of 18 symptoms, frequently associated to chemotherapy and/or immunotherapy treatments, were also graded and recorded. These symptoms included sore throat, dyspnea, fever, cough, chills, vomiting, anosmia, dysgeusia, musculoskeletal pain, abdominal pain, loss of appetite, diarrhea, dizziness, tiredness, expectoration, runny nose, nausea, and headache.

Anthropometric measurements were performed to analyze basal metabolism and nutritional status. The metabolic and nutritional status of patients over time -from baseline V1 (treatment onset as the reference category) to V4 (nine weeks of intervention) and V6 (sixteen weeks of intervention)- were assessed by trained nutritionists using standard validated techniques. The following parameters were recorded: weight (kg), height (cm), arm circumference (cm), triceps skinfold (cm), Body Mass Index (BMI = Weight/Height²), basal metabolic rate (kcal), impedance (Ω), fat mass (percentage and kg), muscle mass (percentage and kg), and total body water (percentage). Body weight was measured using the Body Composition Monitor analyzer (BF511-OMRON HEALTHCARE UK, LT, Kyoto, Japan). Height was measured with a millimeter-precision stadiometer (Leicester Stadimeter-Biológica Tecnología Médica SL, Barcelona). BMI was calculated according to the Quetelet Index formula (BMI = Weight/Height²) (25). Measurements of the triceps skinfold, arm circumference, and arm muscle circumference were taken using a flexible metal tape (range 0-150 cm). Bioelectrical Impedance Analysis (BIA) was performed to measure body fat, muscle mass, and body water using the Body Composition Monitor analyzer (BF511-OMRON HEALTHCARE UK, LT, Kyoto, Japan).

Hemogram, lipid and glucose profiles, and inflammation-associated biomarkers were evaluated through the analysis of a total of 63 specific clinical determinations, including toxicity biomarkers, over time. Patients were instructed to fast overnight before each blood collection. Blood samples were collected in heparinized tubes (BD Vacutainer, Franklin Lakes, NJ, USA) at each visit between 08:00 and 10:00 to minimize circadian variations.

The blood parameters analyzed included: leukocytes, neutrophils, lymphocytes, monocytes, eosinophils, basophils, large unstained cells (LUC), red blood cells (RBCs), hemoglobin, hematocrit, mean corpuscular volume, hemoglobin concentration, red cell distribution width (RDW), platelets, platelet volume, prothrombin time, prothrombin activity, International Normalized Ratio (INR), activated partial thromboplastin clotting time (aPTT), fibrinogen, D-dimer, glucose, urea, creatinine, albumin, calcium, sodium, potassium, chloride, magnesium, total bilirubin, aspartate aminotransferase (AST/GOT), gamma-glutamyl transferase (GGT), alanine aminotransferase (ALT/GPT), alkaline phosphatase (AP), uric acid, cholesterol, HDL-c, LDL-c, triglycerides, total proteins, ionic calcium, iron, creatine phosphokinase (CPK), lactate dehydrogenase (LDH), estimated glomerular filtration rate (eGFR), hemoglobin A1C (IFCC and NGSP), ferritin, transferrin, transferrin saturation index, and C-reactive protein (CRP).

### Multiplex flow cytometry analysis for the immune phenotyping of subpopulations of PBMCs

Peripheral blood samples were collected in TransFix/EDTA Vacuum Blood Collection Tubes (Cytomark, Buckingham, U.K.) to stabilize blood at the point of collection and preserve whole blood specimens until the day of staining and cell acquisition. PBMC isolation was carried out under sterile conditions to avoid monocyte activation. Briefly, whole blood was diluted (1:1) with phosphate buffer solution (PBS) and centrifuged by density gradient with Histopaque-1077 (Sigma–Aldrich, Madrid, Spain) according to the manufacturer’s instructions. After collection, PBMCs were washed twice with PBS.

The cytometry panel allowed for the examination of the frequency of neutrophils, monocytes, lymphocytes, B cells, NK cells, CD4 and CD8 T cells, and natural regulatory T cells (nTregs). Additional markers enabled the characterization of T cell subsets -naïve, memory, effector, and effector memory re-expressing CD45RA (TEMRA)- along with the activation markers CD28 and CD69, and the regulatory markers PD-1 (CD279) and CD25 in the CD4^+^ and CD8^+^ compartments.

In brief, at least 2x10^6 human whole blood cells were incubated with the appropriately fluorescently labeled monoclonal antibodies for 15 minutes at room temperature (RT) in the dark, lysed with 2 mL of Pharm Lyse solution (BD Biosciences) for 10 minutes, and centrifuged at 500× g for 5 minutes at RT. The cell pellet was washed with 5 mL of PBS and centrifuged again. Finally, 0.5 mL of PBS was added, and the sample was analyzed by flow cytometry on a twenty-eight color BD FACSymphony SORP flow cytometer (BD Biosciences). When possible, at least 40,000 events of the CD4 population were acquired. Data were analyzed using FlowJo™ v.10 software for Windows. To generate comparable results among patients and over time, the photomultiplier voltages were adjusted to unlabeled lysed whole blood cells to obtain optimal photomultiplier tube (PMT) voltages for the resolution of dim cell populations (26). The target values resulting from the PMT optimization were used for subsequent calibrations to maintain instrument standardization (27).

Regarding the gating strategy, first, the gating in forward scatter height (FSC-H) vs. forward scatter area (FSC-A) was used to remove doublets, while FSC and side scatter (SSC) gating was used to remove debris. Neutrophils and eosinophils were identified in the SSC/CD45 plot, and lymphocytes and monocytes were selected using the SSC vs. CD4 plot due to better discrimination between both populations than SSC/CD45 expression. After that, for the best lymphocyte identification, basophils were removed in the SSC vs. CD45 plot. Next, lymphocyte populations were further segregated into NK cells, T cells, and B cells according to CD19 and CD3 expression. Memory T cells were measured by CD45RO expression. Using the differential expression patterns of CD45RO and CCR7 (CD197), naïve (CCR7^+^CD45RO^-^), central memory (CCR7^+^CD45RO^+^), effector memory (CCR7^-^CD45RO^+^), and effector memory-expressing CD45RA (CCR7^-^CD45RO^-^) were identified for both CD4 and CD8 T cell populations. Within the CD4^+^ T cells, nTregs were classified by high CD25 expression and low/negative CD127 expression. Additionally, CD279 (PD1), CD25, CD69, and CD28 antigen expression were measured in both CD4 and CD8 T cell populations. **Supplementary Figure 1** shows an example of the gating strategy, and **Supplementary Table 1** summarizes the list of flow cytometer cluster of differentiation (CD) markers used in the study.

### Statistical analysis

#### Assumptions of the analysis

Descriptive analyses of the baseline data were performed by computing the absolute and relative frequencies for categorical variables, and the mean and standard deviation (SD), standard error of the mean (SEM), or 95% confidence intervals (CI) for quantitative variables, depending on the data distribution. To check the homogeneity of the two treatment groups, chi-square or Fisher’s exact tests were used for categorical variables, and Student’s t-test (parametric) or Mann-Whitney U or Wilcoxon tests (non-parametric) were used for quantitative variables.

In this longitudinal study, mixed models were employed to analyze the repeated measurements taken from participants over time (28). The choice of mixed models was driven by their ability to effectively handle the correlated data arising from multiple observations for each individual. By incorporating both fixed effects, which account for population-level effects, and random effects, which capture individual variability, the models addressed potential violations of the independence assumption inherent in traditional statistical methods.

Missing data were handled by allowing mixed models to account for missing values in the outcome variables, as they are robust to missing data under the assumption of data being missing at random (29). This approach ensures that all available data points are used without the need for listwise deletion, preserving statistical power. All variables included in the analysis had less than 20% missing data, minimizing potential bias. Consequently, no additional methods for imputing missing values were required, maintaining the integrity of the analysis.

Furthermore, the inclusion of interaction terms within the mixed models was essential for examining the effect modification between treatment adherence and other covariates. By assessing these interactions, the models provided insights into how the relationship between the treatment and the outcome may vary across different levels of the covariates, such as individual characteristics or time points. This approach allowed for a more nuanced understanding of the dynamics at play, enabling the identification of specific subgroups that may respond differently to the intervention. Overall, the use of mixed models with interaction terms enhanced the reliability and generalizability of the findings in this longitudinal analysis, offering a comprehensive view of the factors influencing treatment outcomes over time.

All predictive models were adjusted for age and sex. Associations were considered significant at a two-tailed test p-value <0.05. All statistical analyses were performed with STATA Statistical Software (version 18.0, College Station, TX, USA; Stata Corp LLC).

#### Analysis of anthropometry measurements

The linear trend of the anthropometric items was evaluated using a linear mixed model to account for repeated measures over time and to assess individual patient variability. This approach provided robust estimates across different time points for metastatic and non-metastatic CRC, BC, and LC treated patients compared to the control placebo treated group. Product terms within the mixed linear models were employed to evaluate potential interactions (effect modification) between treatment adherence and individual anthropometric items.

#### Analysis of the health-related-quality-of-life questionnaire (SF36)

A linear mixed effects model was employed to examine the temporal dynamics of the physical and emotional dimensions of SF36 between Lipchronic^®^ treated patients and control placebo treated patients from baseline V1 (treatment onset as the reference category) to V4 (nine weeks of intervention) and V6 (sixteen weeks of intervention). Additionally, subgroup analyses were conducted based on the type of tumor, including colorectal cancer (CRC), breast cancer (BC), and lung cancer (LC), as well as among metastatic patients categorized as metastatic CRC (mCRC), metastatic BC (mBC), and metastatic LC (mLC). The likelihood ratio test was utilized to ascertain statistical significance. Furthermore, product terms within the mixed linear models were employed to evaluate potential interactions (effect modification) between treatment adherence and immune response.

#### Analysis of symptoms

The linear trend of the symptom’s evolutions was evaluated using a multilevel mixed-effects logistic regression model for the metastatic and non-metastatic CRC, BC and LC treated patients vs. the control placebo treated ones. Product-terms within the mixed linear logistic regression models were employed to evaluate potential interactions (effect modification) between the treatment adherence and individual symptoms.

#### Analysis of inflammation associated biomarkers

Reactive C-protein (CRP), ferritin, transferrin negative reagent, and the inflammatory index neutrophil-to-lymphocyte ratio (NLR) were evaluated to assess the evolution of inflammation in patients during the follow-up period, from baseline V1 (pre-treatment as the reference category) to V6 (end of treatment) and V7 (post-treatment). To assess the adjusted mean linear trend of the clinical determinations, a mixed linear model for the inflammation parameters was applied both for stratified CRC, BC, or LC patients treated with Lipchronic^®^ (Lip) vs. the control placebo (Pla) group, and for the stratified metastatic mCRC, mLC, and mBC patients. Product terms within the mixed linear models were employed to evaluate potential interactions (effect modification) between treatment adherence and inflammation parameters.

#### Multiplex flow cytometry analysis of subpopulations of PBMCs

A mixed linear model was used to test the differential evolution of cytometry parameters over time -baseline V1 (treatment onset as the reference category), V4 (nine weeks), and V6 (sixteen weeks)- for Lipchronic^®^ (Lip) treated patients (CRC, BC, and LC) compared to the control placebo (Pla) group. This analysis was also conducted for stratified metastatic patients. Product terms within the mixed linear models were employed to evaluate potential interactions (effect modification) between treatment adherence and immunity.

#### Analysis of hematologic and biochemical parameters

The linear trend of the clinical determinations was evaluated using a mixed linear model for Lipchronic^®^ (Lip) treated patients (CRC, BC, and LC) compared to the control placebo (Pla) group, as well as for stratified metastatic patients. Product terms within the mixed linear models were employed to evaluate potential interactions (effect modification) between treatment adherence and specific clinical determinations and toxicity parameters. Stratified analyses were applied, considering the limits of normality for each toxicity parameter.

## Results and discussion

### Intervention and study workflow

Rosemary supercritical extracts (SFRE) have been extensively shown to exert anti-inflammatory effects *in vitro*, in preclinical models, and in clinical trials (11, 30). Moreover, the inhibition of oncogenic pathways has been associated with bioactive compounds present in SFRE -mainly the phenolic diterpenes carnosic acid and carnosol- *in vitro* and in preclinical models. One of the main challenges for the application of bioactive extracts in clinical trials is the poor bioavailability of these compounds after gastrointestinal digestion. To address this, SFRE was formulated with bioactive alkylglycerols to enhance the bioavailability of its bioactive compounds (31). This patented formulation Lipchronic^®^ was previously evaluated in a six-week, double-blind, randomized, and parallel pilot study in healthy volunteers. This study confirmed the safety and tolerability of the intervention and revealed immune-metabolic effects that may support the immune response towards effector anti-tumor cells. The ex vivo LPS-stimulation of PBMCs isolated from healthy volunteers indicated an anti-inflammatory cytokine profile associated with Lipchronic^®^ (Lip) intervention compared to the control placebo (Pla).

In this study, we aimed to investigate the functional and immune-metabolic effects of Lipchronic^®^ in cancer patients undergoing clinical treatments. This included evaluating its impact on systemic inflammation, quality of life, symptoms, anthropometry, biochemistry, and tolerability, as well as performing deep immune phenotyping of peripheral blood mononuclear cells (PBMCs) to identify the functional effects on key mediators of the immune system.

### Patient recruitment and study workflow

A total of 108 cancer patients receiving treatments were recruited from November 27, 2020, to March 24, 2021. Clinical and pathological data were collected from medical reports.

Seven visits were conducted: baseline V1 (treatment onset as the reference category), V4 (nine weeks of intervention), V6 (sixteen weeks of intervention), and V7 (twelve weeks post-treatment). Intermediate visits V2, V3, and V5 were performed as control visits. Control visits in our clinical trial refer to routine follow-up appointments that are part of the standard care for oncology patients.

**Figure 1:**
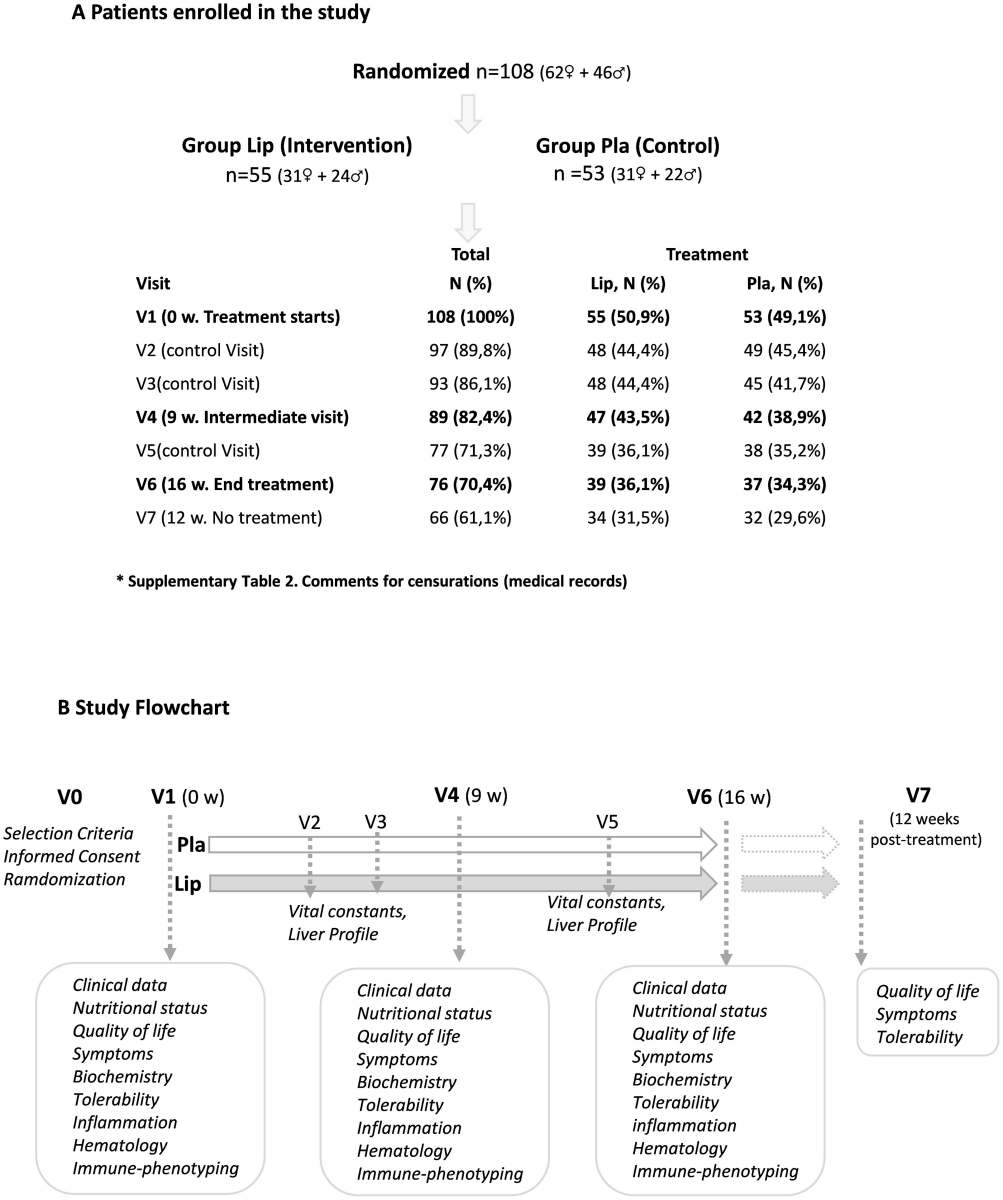
**(A)** Number of patients enrolled in the clinical trial at V0, and censored patients during visits V2, V3, V4, V5, and V6 based on medical records (comments are indicated in **Supplementary Table 2**). **(B)** Study workflow with the main objectives of the study. V1 (initial point); V2, V3, and V5 are control visits; V4 (12 weeks of intervention) and V6 (16 weeks of intervention). V7 (12 weeks after intervention without treatment).

**Figure 1A** summarizes the number of patients enrolled in the clinical trial (V1) and censored patients at visits V2, V3, V4, V5, and V6 based on medical records (comments for censored patients are indicated in **Supplementary Table 2**). V7 corresponds to the visit twelve weeks post-intervention. **Figure 1B** shows the study workflow and primary objectives of the study: V1 (initial point); V2, V3, and V5 are control visits; V4 (nine weeks of intervention); and V6 (sixteen weeks of intervention).

After randomization, no significant differences were observed in baseline characteristics such as age, BMI, sex, tumor type, tumor staging, or treatment, indicating homogeneity between the two groups. **Table 1** summarizes these demographic and baseline characteristics, including the number of cancer patients recruited at V1, categorized by colorectal cancer (CRC), breast cancer (BC), and lung cancer (LC). The percentage of metastatic cancer patients is also indicated. Abbreviations: Lip: Lipchronic^©^; Pla: placebo.

**Table 1:** Descriptive variables (age, BMI and sex), and number of cancer patients recruited (V1) grouped in CRC, breast cancer and lung cancer.

Descriptive variables at beginning of treatment (V1)				
		Lip	Pla	
N	108	55	53	*p-*value
Sex (%)				0,823
*Men*	46 (42,6%)	24 (43,6%)	22 (41,5%)	
*Women*	62 (57,4%)	31 (56,3%)	31 (58,4%)	
Age (y)*	63,7 (11,4)	64,3 (10,9)	63,2 (12)	0,603
BMI *	26,7 (5)	26,6 (5,6)	26,9 (4,5)	
Type of tumor (%)				0,712
*Colorectal*	39 (36,1%)	18 (32,7%)	21 (39,6%)	
* Metastatic (%)*	32 (29,6%)	16 (29,1%)	16 (30,2%)	
*Breast*	27 (25%)	13 (23,6%)	14 (26,4%)	
* Metastatic (%)*	13 (23,6%)	6 (10,9%)	7 (13,2%)	
*Lung*	19 (17,6%)	10 (18,2%)	9 (17%)	
* Metastatic (%)*	16 (29,1%)	8 (14,5%)	8 (15,1%)	
*Others*	23 (21,3%)	14 (25,5%)	9 (17%)	
* Metastatic (%)*	13 (23,6%)	5 (9,1%)	8 (15,1%)	
*Tumor extension [N (%)]*	108	55	53	0.499
*Local*	23	13 (23.64%)	10 (18.87%)	
*Metastatic*	74	35 (63.64%)	39 (73.58%)	
*Regional*	11	7 (12.73%)	4 (7.55%)	
*Treatment*	108			0.502
*Advanced disease*	73	37 (67.27%)	36 (67.92%)	
*Adjuvance*	20	12 (21.82%)	8 (15.09%)	
*Neoadjuvance*	15	6 (10.91%)	9 (16.98%)	

*p*-value calculated by t-Student for the continuous variables, and Chi for the categorical variables.

*Mean (SD). Percentage of metastatic cancer patients are also indicated. Lip, Lipchronic intervention group; Pla, placebo control group.

The mean age was comparable between the two groups, with a mean (+SD) of 64.3 (+10.9) years in the Lipchronic^®^ group and 63.2 (+12) years in the placebo group (p-value = 0.603). The BMI values were similarly consistent, with a mean (SD) of 26.6 (+5.6) in the Lipchronic^®^ group and 26.9 (+4.5) in the placebo group. Sex distribution was balanced, with 43.6% men in the Lipchronic^®^ group and 41.5% in the placebo group, while 56.3% and 58.4%, respectively, were women (p-value = 0.823). Tumor type distribution showed no significant differences, with 32.7% of patients in the Lipchronic^®^ group and 39.6% in the placebo group having CRC, 23.6% and 26.4% having BC, and 18.2% and 17% having LC, respectively (p-value = 0.712). Additionally, metastatic disease was present in 63.64% of the Lipchronic^®^ group and 73.58% of the placebo group (p-value = 0.499), further reinforcing the comparability between groups and supporting the robustness of the study’s randomization.

### Safety and tolerability

As expected for patients with advanced cancer under active treatments, this was a very symptomatic population. However, no clinical adverse effects attributable to the supplement were detected during the study. Tolerance and toxicity after the intervention were evaluated through clinical and full hematological and biochemical monitoring.

The only statistically significant difference found in the interaction treatment x visit was a mild increase in the liver AST/GOT enzyme concentration at V4 (p-value = 0.049) with Lipchronic^®^. Nevertheless, the stratified analysis considering the normal laboratory values for this parameter remained within normal ranges. AST/GOT differences were minor, and no other hepatic alterations were detected in the general population of cancer patients. Additionally, a marginal effect in the interaction treatment x visit was observed for the ALT parameter (p-value = 0.066) without statistically significant differences between groups. In the subgroup of colorectal cancer patients, a marginal effect in the interaction treatment x visit was also observed for the ALT parameter (p-value = 0.058) without statistically significant differences between groups. One breast cancer patient receiving treatment with ribociclib showed grade 4 transient elevation of AST and ALT. Although liver toxicity can develop with ribociclib, Lipchronic^®^ was suspended. Another patient previously received this combination without toxicity. Since cytochrome P interactions cannot be discarded, it seems reasonable to avoid combining rosemary and other herbal extracts with drugs metabolized by the liver outside of a clinical trial.

### Symptoms and impact on quality-of-life parameters assessed by the SF36 questionnaire

A total of 18 symptoms frequently associated with chemotherapy and/or immunotherapy treatments were graded and recorded. These symptoms included sore throat, dyspnea, fever, cough, chills, vomiting, anosmia, dysgeusia, musculoskeletal pain, abdominal pain, loss of appetite, diarrhea, dizziness, tiredness, expectoration, runny nose, nausea, and headache. No differences were found in the comparison of Lip and Pla intervention groups, except for a significant decrease in anosmia in Lip group of cancer patients (p-value = 0.032 at V4, and p-value = 0.024 at V6), and a tendency towards the reduction of dysgeusia in Lip group compared to Pla group of cancer patients (p-value = 0.061 at V6). Nevertheless, these results should be considered with caution as only 6 to 2 patients were positive for anosmia and dysgeusia.

The effect of the intervention on quality of life was evaluated with the SF36 questionnaire, composed of 36 questions (items) to evaluate both positive and negative states of the patient’s health, considering both physical and mental dimensions. Despite not being specific, the SF36 questionnaire is widely used for measuring the health status of cancer patients. For each parameter, scores were coded, summed, and transformed to a scale from 0 (the worst possible condition) to 100 (the best possible condition).

No statistically significant differences were observed in the interaction treatment x visit, except for the SF36 vitality item, where a tendency towards an improved score was observed in the Lip group of cancer patients at V6 (p-value = 0.029), not distinguished by the type of tumor. When stratified by the type of tumor, a statistically significant interaction treatment x visit was found in the LC subgroup of cancer patients (p-value = 0.021), where a tendency towards an increased score on vitality was associated with Lip compared to Pla intervention (p-value = 0.056 at V6) (**Supplementary Table 3**).

### Anthropometry and plasma lipid and glucose profiles

Anthropometric measurements were collected by specialists from the Hospital Universitario Infanta Sofía in collaboration with dieticians specialized in oncology from the IMDEA Food Institute. To evaluate the effects of the intervention on anthropometry, nine parameters-weight (Tanita^®^), height, arm circumference, triceps perimeter, BMI, impedance, basal metabolism, fat mass (kg), muscle mass (kg), and total water- were recorded over time, from baseline V1 (pre-treatment as the reference category) to V4 (nine weeks of treatment, intermediate visit) and V6 (sixteen weeks of treatment, end of treatment). Comparisons were made considering all cancer patients receiving Lip vs. Pla, as well as stratified by tumor type -colorectal cancer (CRC), breast cancer (BC), and lung cancer (LC). When possible, the evolution of the anthropometric parameters in metastatic patients (mLC, mCRC, mBC) was also compared.

**Figure 2:**
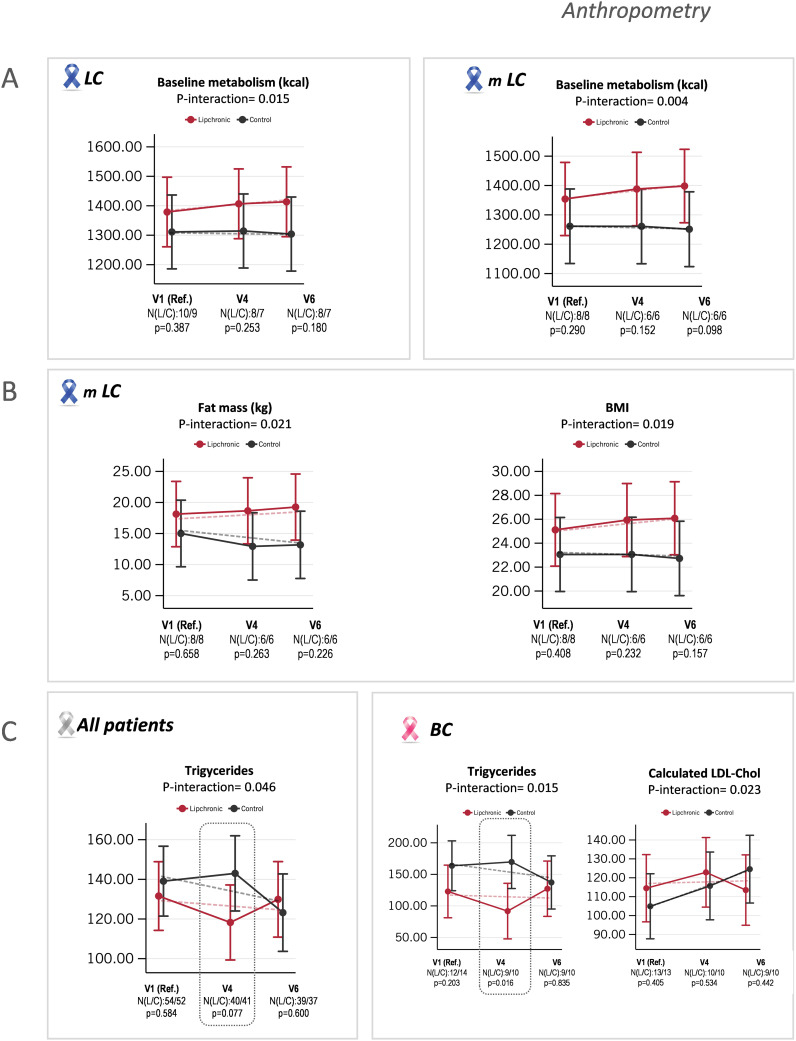
Indicates the age- and sex-adjusted means with 95% confidence intervals (CI) at V1, V4, and V6, as well as the linear predictions for anthropometric parameters and lipid profile. **(A)** Statistically significant differences found in the interaction treatment and visit for baseline metabolism (kcal) between Lipchronic^®^ (Lip) and control placebo (Pla) in the lung cancer (LC) group and in the metastatic lung cancer (mLC) subgroup. **(B)** Statistically significant differences found in the interaction treatment and visit for fat mass (kg) and BMI between Lip and control Pla in the mLC subgroup of patients. **(C)** Statistically significant differences found in the interaction treatment and visit for total plasma triglycerides between Lip and control Pla groups, in all cancer patients not stratified by tumor type. **(D)** Statistically significant differences observed in the interaction treatment and visit in the breast cancer (BC) group and in the stratified metastatic breast cancer (mBC) subgroup.

No statistical differences were observed when comparing Lip vs. control Pla intervention in cancer patients not stratified by tumor type. However, differences were found when patients were stratified by tumor type. Statistically significant differences were observed in the interaction treatment x visit in the LC subgroup of patients in basal metabolism (p-value = 0.015; N Lip/Pla: 10/9), where a tendency to increase in the Lip LC group compared to the control Pla LC group was observed. These effects were especially striking in the stratified subgroup of metastatic LC (mLC) patients, where statistically significant differences in the interaction treatment x visit were found in basal metabolism (p-value = 0.004; N Lip/Pla: 8/8) (**Figure 2A**). Additionally, statistically significant differences were observed in the interaction treatment x visit in the mLC subgroup in fat mass (p-value = 0.0021; N Lip/Pla: 8/8) and BMI (p-value = 0.0019; N Lip/Pla: 8/8). In all cases, a tendency to increase in the Lip mLC group compared to the control Pla mLC group was found. **Figure 2A** shows mean adjusted by age and sex of the linear predictions for basal metabolism (kcal) between Lip LC vs. control Pla LC (left panel), and between Lip mLC vs. control Pla mLC (right panel). **Figure 2B** shows mean adjusted by age and sex of the linear predictions for BMI and fat mass (kg) between Lip mLC vs. control Pla mLC.

Regarding lipid and glucose profiles, statistically significant differences in the interaction treatment x visit for triglyceride levels were found when all cancer patients, not stratified by tumor type, were compared (p-value = 0.046; N Lip/Pla: 54/52), with a marginal tendency to decrease in the Lip group compared to the control Pla group at V4 (p-value = 0.077; N Lip/Pla: 40/41) (**Figure 2C**). Considering the peculiarities of the distinct types of cancers -where LC patients frequently undergo progressive malnutrition, CRC patients associate with local and systemic inflammation, and localized BC patients are prone to develop metabolic syndrome over time- we next performed the analysis in cancer patients stratified by tumor type (LC, BC, and CRC subgroups). Statistically significant differences were found only in the BC subgroup, where a significant decrease in triglyceride levels was observed in the Lip group vs. the control Pla group, suggesting this group contributed to the global effect observed for triglyceride reduction (p-value = 0.016 at V4, N Lip/Pla: 9/10). Additionally, the calculated LDL-cholesterol parameter was stabilized in the Lip BC subgroup compared to the control BC subgroup, where a tendency to increase was found. When the analysis was done considering only the metastatic BC subgroup, no effects were observed. These results should be taken with caution because although controlling metabolic syndrome in non-disseminated BC is desirable, the opposite occurs in disseminated BC patients.

### Hemogram and inflammation-associated biomarkers

**Figure 3:**
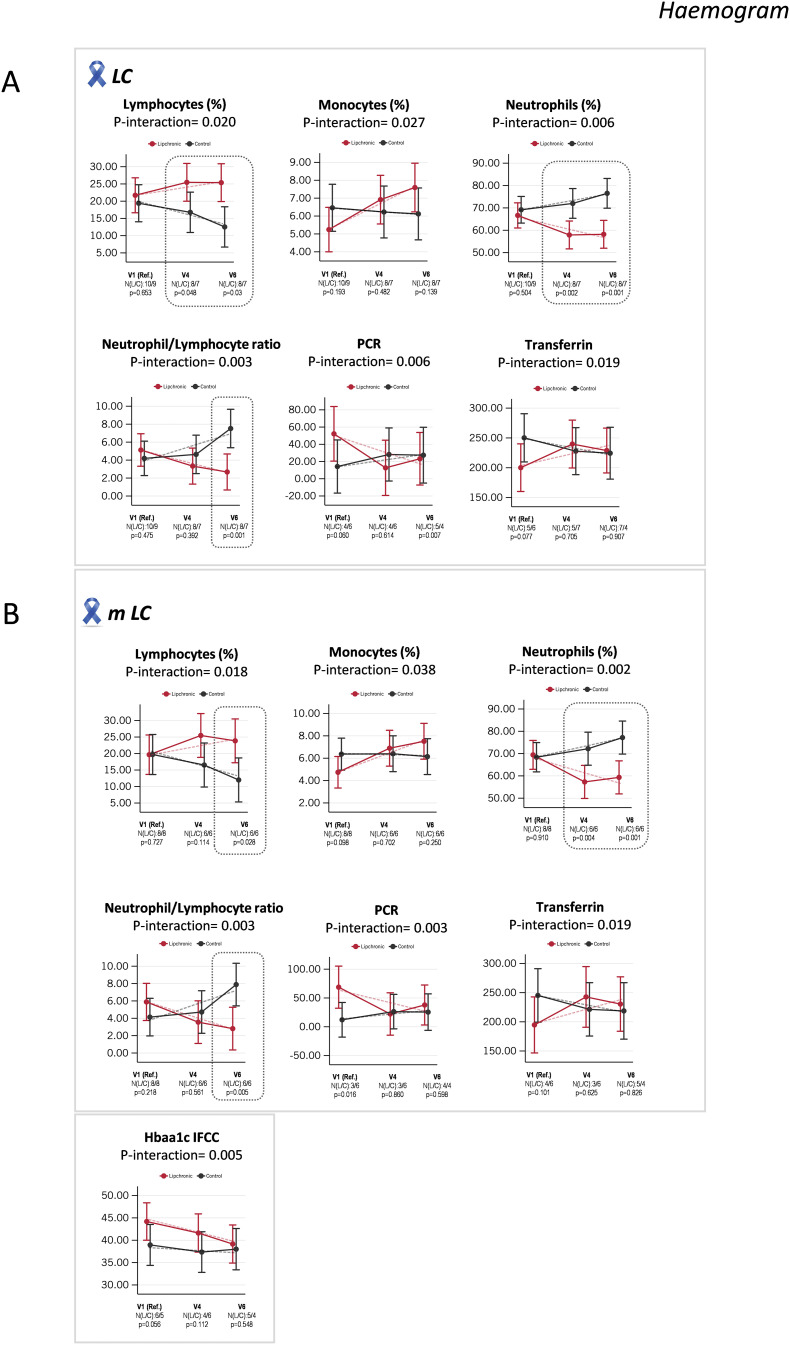
Indicates the age- and sex-adjusted means with 95% confidence intervals (CI) at V1, V4, and V6, as well as the linear predictions for hemogram parameters. **(A)** Statistically significant differences found in the interaction treatment and visit for lymphocytes (%), monocytes (%), neutrophils (%), NLR, CRP and transferrin between Lip and control Pla in the LC group; and **(B)** in the interaction treatment and visit for lymphocytes (%), monocytes (%), neutrophils (%), NLR, CRP, transferrin, and HbA1c-IFCC, between Lip and control Pla in the stratified mLC subgroup. NLR, Neutrophil-to-lymphocyte ratio; CRP, C-reactive protein, HbA1c, Glycated hemoglobin; IFCC; International Federation of Clinical Chemistry and Laboratory Medicine.

Comparisons of the hemogram profile revealed statistically significant differences in the interaction treatment x time in the LC subgroup of patients (**Figure 3**). Importantly, the percentage of lymphocytes was statistically increased in the Lip LC group compared to the control Pla group (p-value = 0.048, N Lip/Pla: 8/7, at V4; p-value = 0.003, N Lip/Pla: 8/7, at V6). Conversely, the percentage of neutrophils was statistically diminished in the Lip LC group (p-value = 0.002, N Lip/Pla: 8/7, at V4; p-value = 0.001, N Lip/Pla: 8/7, at V6) compared to the control Pla LC group, where a tendency to increase was found (**Figure 3A**). Similar results were found in the metastatic subgroup of LC patients, where lymphocytes were statistically higher in the Lip mLC group compared to the control Pla mLC group (p = 0.028, N Lip/Pla: 6/6, at V4); meanwhile, the percentage of neutrophils was statistically diminished in the Lip mLC group (p-value = 0.004, N Lip/Pla: 6/6) at V4, and at V6 (p-value = 0.001, N Lip/Pla: 6/6) (**Figure 3B**).

Of relevance, the inflammatory index of neutrophil-to-lymphocyte ratio (NLR) was reduced in the Lip group compared to the control Pla group at V6, both in the LC group of patients (p-value = 0.001, N Lip/Pla: 8/7) and in the mLC subgroup of patients (p-value = 0.005, N Lip/Pla: 6/6) (**Figures 3A, B**). These results suggest that Lip intervention may be associated with better control of systemic inflammation, in line with the tendency towards the reduction of C-reactive protein levels from V1 to V6 in the Lip subgroup of patients. Recently, a lung immune prognostic index (LIPI), calculated from the ratios of derived neutrophil-to-lymphocyte (dNLR) and lactate dehydrogenase (LDH) levels, has been identified as a prognostic factor for NSCLC patients receiving targeted therapy, chemotherapy, and immunotherapy (32, 33).

Additionally, the total levels of transferrin were maintained in the Lip LC group compared to the control Pla LC group, where a tendency to diminish from V1 to V6 was found (**Figure 3A**). Since inflammation is a key promoter of the inhibition of erythropoiesis, these results suggest that Lip intervention may protect LC patients from anemia, which is frequently associated with patients undergoing clinical treatments (34).

No relevant effects were found on systemic glucose homeostasis, except for the glycated hemoglobin biomarker (HbA1c IFCC), which showed a tendency to diminish in the Lip mLC subgroup of patients from V1 to V6, while in the control Pla LC subgroup, this parameter was maintained (**Figure 3B**). This result may indicate better nutrient utilization associated with Lip intervention compared to Pla in the mLC subgroup of patients, who frequently suffer from malnutrition.

### Immune phenotyping of PBMCs

The immune system’s control of tumors, or immune surveillance, depends on the type of cancer, genetic and molecular alterations, stage, and other individual factors such as the patient’s overall health. Cancer cells frequently develop mechanisms to evade immune system detection and destruction, leading to tumor growth and/or dissemination. This has strengthened interest in developing strategies to enhance the immune system’s ability to inhibit tumor dissemination, a field known as cancer immunotherapy. Although several approaches are used to harness the immune system’s power in cancer -checkpoint inhibitors, CAR-T cell therapy, cancer vaccines, cytokines- immune therapy is not equally effective for all types of cancers or all patients.

Increased immune capabilities and reduced inflammatory responses have historically been observed in individuals with a healthy lifestyle and nutritional habits. Recently, an exploratory in silico cross-sectional analysis using an advanced machine learning model from transcriptomic and proteomic data of nearly 7,500 oncologic patients in three major cohorts, including lung cancer (LC), colorectal cancer (CRC), and breast cancer (BC), and the reported mechanism of action for extracts from Rosmarinus officinalis L., revealed a high correlation between the molecular triggering points in cancer (MTPs) -mainly inflammation and immune system constriction- and the molecular effects of a registered bioactive formula based on diterpenic phenols from rosemary, suggesting clinical benefits of the bioactive formula as a coadjuvant in the clinical treatment of cancer patients (23).

### Immune phenotyping of PBMCs

To elucidate the potential functional effects of Lip on key mediators of the immune system, we performed a complete immune phenotyping of PBMC subpopulations using multiplex FACS analysis.

**Figure 4:**
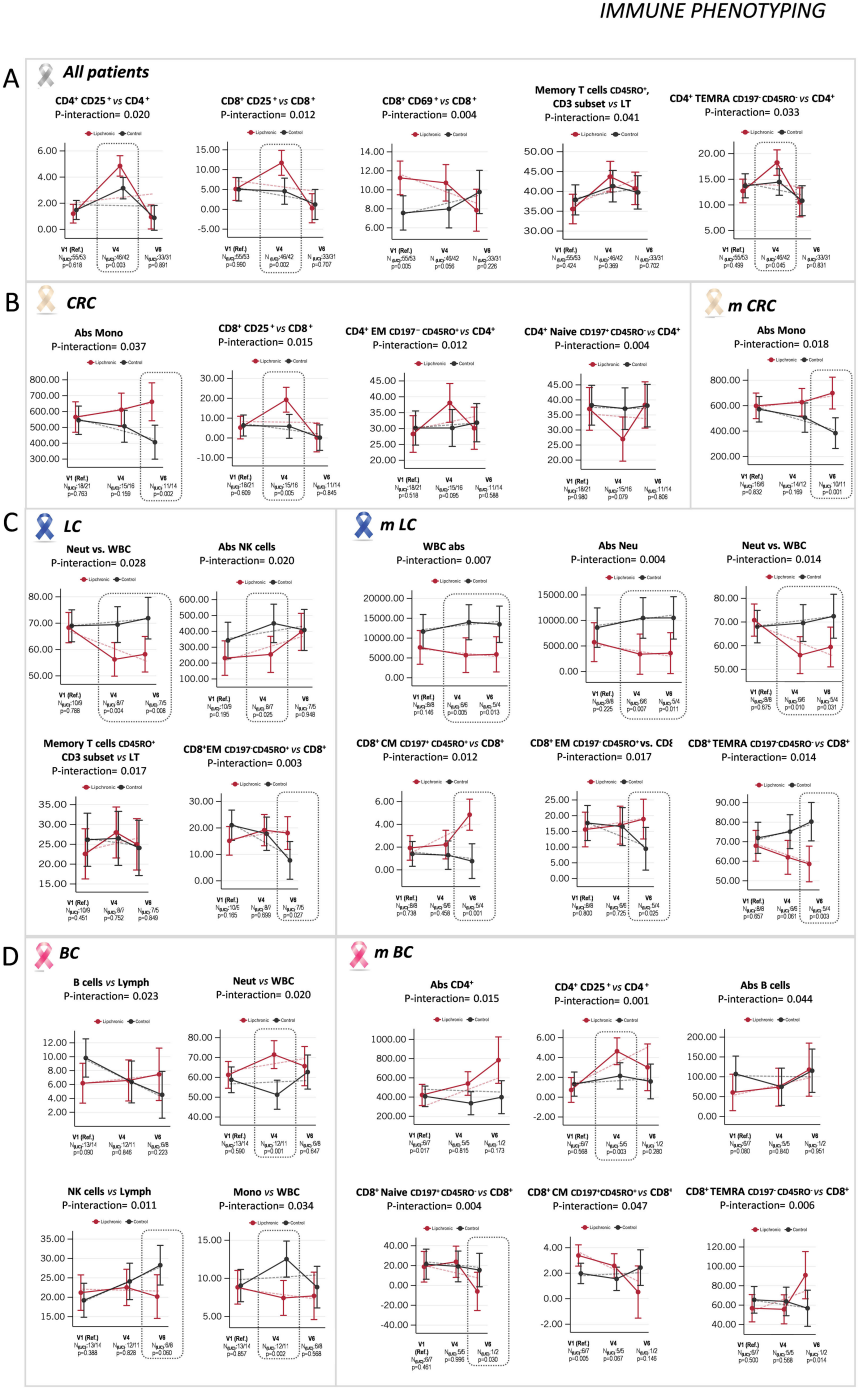
Indicates the age- and sex-adjusted means with 95% confidence intervals **(CI)** at V1, V4, and V6, as well as the linear predictions for PBMC subpopulations. **(A)** Statistically significant differences found in the interaction treatment and visit for the indicated subpopulations between Lip and control Pla groups, not stratified by tumor type. **(B)** Significant differences observed in the interaction between treatment and visit for the indicated subpopulations between Lip and control Pla in the colorectal cancer (CRC) group, and in the metastatic colorectal cancer (mCRC) subgroup; **(C)** in the LC group and in the mLC subgroup; and **(D)** in the BC group, and in the mBC subgroup.

First, we analyzed the effects of Lip vs. control Pla in cancer patients not stratified by tumor type. **Figure 4A** shows all the statistically significant differences found in the interaction treatment and visit for Lip and control Pla groups, indicating the age- and sex-adjusted means with 95% confidence intervals (CI) at V1, V4, and V6, together with the linear predictions for the indicated PBMC subpopulations. Notably, a significant increase in the pools of CD4^+^CD25^+^ (p-value = 0.003, N Lip/Pla: 46/42) and CD8^+^CD25^+^ (p-value = 0.002, N Lip/Pla: 46/42) at V4 was found in the Lip group vs. the control Pla group, with no differences observed at V6. These results suggest an earlier activation and, consequently, a higher potential effector activity for the CD4 and CD8 pools associated with Lip in cancer patients not stratified by tumor type. Importantly, both populations returned to basal levels at V6. Additionally, a tendency to reduce the CD69^+^ marker, an early activation marker upon TCR receptor activation, specifically in the CD8^+^ subpopulation, was found only in the Lip group of cancer patients from V1 to V6. Overall, these results suggest that Lip treatment sustains a rapid activation of the T cell response (**Figure 4A**).

A marginal tendency to increase the memory CD45RO^+^ lymphocytes relative to the total CD3^+^ lymphocytes in the Lip group vs. the control Pla group in cancer patients not stratified by tumor type was found. This is important as the preservation of memory T cell subpopulations is crucial for mounting effective immune responses in the crosstalk between the lymph nodes and peripheral circulation. Finally, the TEMRA effector memory subpopulation relative to the total CD3 lymphocytes was augmented in the Lip group of cancer patients vs. the control Pla group at V4 (p-value = 0.045, N Lip/Pla: 46/42), suggesting a higher capacity to maintain the terminally differentiated effector memory cells, known to have higher plasticity as they re-express the CD45RA naïve marker (35). Nevertheless, basal levels were restored at V6. Complex variables are involved in past immune responses, allowing some degree of heterogeneity in the TEMRA subpopulation. Several studies have demonstrated the existence of TEMRA cells expressing CD27^+^CD28^+^ concomitant with low expression levels of KLRG1 and CD57 (markers of senescence and exhaustion, respectively), suggesting a relatively less differentiated state compared to CD27^−^CD28^−^ TEMRA cells (36).

Considering that oncogenic alterations associated with tumor type affect anti-tumor immune responses, we performed similar analyses in stratified CRC, BC, and LC; and in stratified metastatic mCRC, mBC, and mLC.

### Effect of Lip on PBMC subsets in CRC patients

**Figure 4B** shows all the statistically significant differences found in the interaction treatment and visit for the CRC group of cancer patients, indicating the age- and sex-adjusted means with 95% confidence intervals (CI) at V1, V4, and V6, together with the linear predictions of the indicated PBMC subpopulations. In CRC patients, an increase in absolute monocytes at the late stage (V6) was observed associated with the Lip experimental treatment, while a decrease was found in the control Pla group (p-value = 0.002, N Lip/Pla: 11/14). This effect was also observed when considering only the stratified subgroup of mCRC, where higher absolute values were observed associated with Lip vs. the control Pla group (p-value = 0.001, N Lip/Pla: 10/11) (**Figure 4B**). These results suggest that Lip may preserve this population after clinical treatments.

Additionally, the CD8^+^CD25^+^ population (already activated) increased in the Lip CRC subgroup of patients during the first period (V4) (p-value = 0.005, N Lip/Pla: 15/16), returning to initial levels at V6, suggesting that this population would no longer be activated. Conversely, the control Pla CRC group of patients showed a decrease in this activated CD8 population, which may reflect the toxicity associated with clinical treatments.

A tendency to increase the effector memory CD4 T lymphocytes (CD4^+^CD197-CD45RO^+^), especially at V4 (p-value = 0.095, N Lip/Pla: 15/16) in the Lip group vs. the control Pla group, was observed, in line with the tendency to decrease the corresponding naïve CD4 population (p-value = 0.079, N Lip/Pla: 15/16) (**Figure 4B**), suggesting the maintenance of cytotoxic activity associated with Lip intervention.

Interestingly, monocytes increased in absolute values only in the late stages (V6) with Lip experimental treatment, and decreased with Pla treatment, both in all CRC patients (p-value = 0.002, N Lip/Pla: 11/14) and in the stratified metastatic subgroup of CRC patients (p-value = 0.001, N Lip/Pla: 10/11) (**Figure 4B**). It is tempting to speculate that Lip may contribute to their preservation after clinical treatments.

### Effect of Lip on PBMC subsets in LC patients

**Figure 4C** shows all the statistically significant differences found in the interaction treatment and visit for the LC group of cancer patients, indicating the age- and sex-adjusted means with 95% confidence intervals (CI) at V1, V4, and V6, together with the linear predictions of the indicated PBMC subpopulations. In LC patients, Lip was associated with an increase in the CD8 effector memory (EM) T cell population (p-value = 0.027; N Lip/Pla: 7/5). Additionally, when the analysis was done considering only the stratified mLC subgroup of LC patients, an increase in the CD8 central memory (CM) (p-value = 0.001, N Lip/Pla: 5/4) and in the CD8 effector memory (EM) (p-value = 0.025, N Lip/Pla: 5/4) T cell subpopulations at V6 was observed. Interestingly, the TEMRA CD8 subpopulation was significantly reduced in the stratified subgroup of mLC vs. the control Pla mLC group (p-value = 0.003, N Lip/Pla: 5/4) at V6. These results suggest an overall preservation of CD8 cytotoxic activity from exhaustion associated with the Lip intervention vs. the control Pla (**Figure 4C**).

The strongest effects were observed in the mLC subgroup of patients, where the experimental treatment Lip was associated with a decrease in the absolute numbers of leukocytes in both study periods (p-value = 0.013, N Lip/Pla: 6/6, at V4; p-value = 0.010, N Lip/Pla: 5/4, at V6). This decrease did not cause leukopenia, as leukocyte counts were maintained above the minimum range of normal counts (normally 4500 leukocytes/mm³). Moreover, this decrease seemed to be at the expense of the absolute neutrophil counts, which decreased in the experimental treatment Lip (p-value = 0.007, N Lip/Pla: 6/6, at V4; p-value = 0.011, N Lip/Pla: 5/4, at V6) vs. the control Pla treatment. Nevertheless, despite this decrease, neutrophils were also within the normal range (>2000 neutrophils/mm³). These results align with the reduction of the inflammatory index NLR previously observed in the Lip-LC and Lip-mLC subgroups of patients vs. the control Pla subgroups (**Figure 2**).

### Effect of Lip on PBMC subsets in BC patients

**Figure 4D** shows all the statistically significant differences found in the interaction treatment and visit for the BC group of cancer patients, indicating the age- and sex-adjusted means with 95% confidence intervals (CI) at V1, V4, and V6, together with the linear predictions of the indicated PBMC subpopulations. In BC patients, Lip was associated with a marginal reduction of the NK subpopulation compared to the control Pla group (p-value = 0.06, N Lip/Pla: 6/8, at V6), while the B lymphocyte population showed a tendency to diminish in the Pla group, suggesting a role of Lip in preserving the humoral response after clinical treatments.

Additionally, Lip augmented the percentage of neutrophils (p-value = 0.001, N Lip/Pla: 12/11) to the detriment of monocytes (p-value = 0.002, N Lip/Pla: 12/11), especially in the late period of the intervention (V6).

The most interesting effects were observed in the stratified subgroup of mBC patients, where Lip was associated with an increase in the activated CD4^+^CD25^+^ T lymphocytes (p-value = 0.03, N Lip/Pla: 5/5, at V4), concomitant with a tendency to augment the absolute counts of CD4^+^ T lymphocytes compared to the control Pla mBC group. Additionally, the naïve CD8 T cell subpopulation was diminished (p-value = 0.030, N Lip/Pla: 12/11), while the CD8^+^ TEMRA T lymphocytes were increased (p-value = 0.014) at V6. These results suggest a role of the activated CD4^+^ population in promoting a functional terminal effector CD8 activated population (i.e., CD8^+^ TEMRA). As previously indicated, some degree of heterogeneity in the TEMRA subpopulation has been shown, where a specific subset of TEMRA cells expressing CD27^+^CD28^+^ concomitant with low expression levels of KLRG1 and CD57 has been associated with higher functional effector activity compared with CD27^−^CD28^−^ TEMRA cells (36). It is tempting to hypothesize that Lip may contribute to maintaining CD8^+^ TEMRA cells with low proliferation capacity but increased cytotoxic activity (i.e., increased perforin and granzyme B), although the status of additional biomarkers, such as CD27, CD28, KLRG1, or CD57, should be evaluated.

## Conclusions

In general, a potentiation of key mediators of the immune system was observed with the experimental treatment Lipchronic^®^ (Lip) compared to the control placebo (Pla) treatment, with specific effects among different patient groups. In Lung Cancer (LC) patients Lip was associated with a decrease in the total number of neutrophils without reaching leukopenia. An increase in the NK population and memory T lymphocytes (both CD8 EM and CD8 CM), especially in metastatic patients, was observed with Lip intervention. In Colorectal Cancer (CRC) patients Lip was associated with the preservation of monocytes and the maintenance of late-activated effector cytotoxic CD8^+^CD25^+^ T lymphocytes, suggesting a preservation of innate and cytotoxic activity. The effector memory (EM) CD4 T lymphocyte subpopulation was preserved, while a decrease in the naïve subset of T cells was associated with Lip. In Breast Cancer (BC) patients Lip seemed to affect the humoral response of B lymphocytes (potentially for antibody production) and increased CD4 T lymphocytes, which was not observed in the control Pla group.

A reduction in biomarkers of systemic inflammation, such as ferritin, C-reactive protein, and the inflammatory index Neutrophil/Lymphocyte ratio (NLR), was observed, especially in the LC subgroup of cancer patients treated with Lip. Additionally, a significant increase in hemoglobin levels was noted, suggesting potential benefits in disease progression and complications.

In the LC group, Lip was associated with increases in body weight, BMI, basal metabolism, and fat mass compared to the control placebo group, indicating potential benefits in disease progression.

From a safety perspective, the supplement was generally well tolerated in these highly medicated patients. Follow-up is recommended for patients with pre-existing liver disease at the start of treatment, as well as vigilance for potential drug interactions.

## Limitation of the study and future directions

The study involved 108 patients, which may be considered a relatively small sample size for drawing broad conclusions. Larger studies are needed to confirm the findings and ensure they are generalizable. In addition, the study was conducted in a specific population (patients from a single hospital in Spain). The generalizability of the findings to other populations and settings might be limited.

While the study stratified patients by tumor type and metastatic status, further stratification based on molecular profiles and specific treatment regimens could provide more detailed insights. The lack of such stratification might limit the understanding of how different subgroups respond to the intervention.

The follow-up period was relatively short (up to 16 weeks of intervention). Longer follow-up periods are necessary to assess the long-term effects and sustainability of the observed benefits.

The study controlled for age and sex, but other potential confounding factors, such as lifestyle, diet, and concurrent medications, might influence the results. Addressing these factors in the analysis could strengthen the findings.

Finally, while the study provided some insights into the immune-modulatory effects of Lipchronic^®^, further mechanistic studies are needed to understand the underlying biological processes and how they contribute to the observed clinical outcomes.

The suggested potentiation of the immune system and anti-inflammatory effects could be of great interest for cancer patients, particularly in combination with immunotherapies. Our results merit further exploration, with a specific focus on lung cancer patients. A randomized clinical trial should be conducted to evaluate the potential synergism of Lipchronic^®^ with immunotherapies.

This study opens the door to new clinical trials in more controlled patient populations. Future studies should consider stratifying patients based on molecular profiles, tumor characteristics, and the treatments they receive. Stratification based on these factors could provide a more detailed understanding of the treatment effects and allow for the personalization of therapies to improve clinical outcomes.

Additionally, it would be beneficial to explore the long-term effects of Lipchronic^®^ on immune modulation and inflammation control in cancer patients. Investigating the molecular mechanisms underlying the observed benefits could also provide insights into how to optimize the use of Lipchronic^®^ in combination with other cancer treatments.

Overall, our findings highlight the potential of molecular nutrition-based strategies as complementary tools in the clinical management of cancer patients, particularly in the current era of novel immunotherapies. Further research in this area could lead to more effective and personalized treatment approaches, ultimately improving patient outcomes.

## Data Availability

The original contributions presented in the study are included in the article/Supplementary Material. Further inquiries can be directed to the corresponding authors.

## Supplementary material

Supplementary Table 1 List of multiparametric flow cytometry markers.

Supplementary Table 2 Comments for censured patients.

Supplementary Table 3 Statistically significant differences in the interaction treatment x visit for quality-of-life parameters assessed by the SF36 questionnaire.

Supplementary Figure 1 Gating strategy for the immune phenotyping of PBMC subpopulations.
