# Supplementary material for: Randomized clinical trial in cancer patients shows immune metabolic effects exerted by formulated bioactive phenolic diterpenes with potential clinical benefits
Source: Front Immunol. 2025 Feb 17;16:1519978. doi: 10.3389/fimmu.2025.1519978 (PMC11872936; doi:10.3389/fimmu.2025.1519978)
Supplement: Supplementary Table 1 — List of multiparametric flow cytometry markers. [file Table1.docx]

**Supplementary Tables**

**Supplementary Table 1**: Antibody panel used for the 12-color immunophenotyping panel

| **Marker** | **Fuorophore** | **Detector Filter** | **Supplier** | **Cat number** | **Clon** |
| --- | --- | --- | --- | --- | --- |
| CD45RO | BUV395 | UV 379/28-A | BD | 564292 | UCHL1 |
| CD8 | BUV496 | UV 515/30-A | BD | 612942 | RPA-T8 |
| CD25 | BUV615 | UV 605/20-A | BD | 612997 | 2A3 |
| CD19 | BUV661 | UV 670/25-A | BD | 612971 | 1D3 |
| CD279 | BV421 | V 431/28-A | BD | 564323 | MIH4 |
| CD45 | BV510 | V 525/50-A | BD | 563204 | HI30 |
| CD28 | BV605 | V 605/40-A | BD | 562976 | CD28.2 |
| CD4 | BV786 | V 810/40-A | BD | 563877 | SK3 |
| CD3 | BB515 | B 530/30-A | BD | 565100 | HIT3a |
| CD197 | BB700 | B 710/50-A | BD | 566437 | 3D12 |
| CD127 | PE | YG 586/15-A | BD | 557938 | HIL-7R-M21 |
| CD69 | PE-CY5 | YG 670/30-A | BD | 555532 | FN50 |

**Supplementary Table 2.** Follow up of number of cancer patients during visits and censured patients based on medical records. Percentage of metastatic cancer patients are indicated. Lip: Lipchronic, intervention group; Pla: placebo, control group.

|  | **Total** | **Treatment** | |
| --- | --- | --- | --- |
| **Visit** | **N (%)** | **Lip, N (%)** | **Pla, N (%)** |
| V1 | 108 (100%) | 55 (50,9%) | 53 (49,1%) |
| V2 | 97 (89,8%) | 48 (44,4%) | 49 (45,4%) |
| V3 | 93 (86,1%) | 48 (44,4%) | 45 (41,7%) |
| V4 | 89 (82,4%) | 47 (43,5%) | 42 (38,9%) |
| V5 | 77 (71,3%) | 39 (36,1%) | 38 (35,2%) |
| V6 | 76 (70,4%) | 39 (36,1%) | 37 (34,3%) |
| V7 | 66 (61,1%) | 34 (31,5%) | 32 (29,6%) |

Censured patients based on medical records

| **Visit** | **Lip** | **Pla** |
| --- | --- | --- |
| V0 | **-** | **-** |
| V1 | *code 84*: took 6 first week and 4 the second, then quit; *code 51*: he has not taken medication; *code 105*: suspends it after 2-3 weeks, delivers nothing; *code 62*: it took 12 days everything, then nothing; *code 99*: take the first 3 capsules and nothing else; *code 100*: take the first 2 capsules and nothing else; *code 14*: does not deliver boxes, it was not possible to contact the family | *code 34*: take 14 capsules in the first 18 days and stop; *code 91*: took the first 3 capsules; *code 63*: it took 12 days everything, then nothing; *code 86*: took 4 the first and 4 the second week, then quits |
| V2 | - | *code 92*: took 7 the first and second week, 3 the third and quit due to mild diarrhea; *code 3*: 12 capsules in 16 days, until dysphagia with one capsule, discontinued, no warning; *code 56*: 21/21 and then stop taking it, leave rehearsal,; *code 9:* the family brings empty v4 questionnaires (the date was not reached), and adherence record (41/42 cp) and blisters |
| V3 | *code 27*: on admission it was asked and it was taken until the day before admission | *code 25*: it took 6 weeks in a row (36/41), record and blisters coincide; *code 37*: everything up to box 2 week 15 and first 2 cp; *code 71*: take everything up to blister 13 first three, then nothing |
| V4 | *code 44*: 8 blister is missing from box 1 but recorded every day and delivered in box 2, then nothing is taken from box 2; *code 6*: take everything but one capsule up to blister 9, end 13/2 says the cap; *code 26*: 6 are missing from blister 8 (week 8); *code 66*: After death, he does not deliver a second box or an inheritance sheet, the end of treatment date is extrapolated by empty blister packs from the first box; *code 52*: took everything up to second blister of second box; *code 28*: took everything up to 5-2 (7 weeks 2 days, 51 caps), visit 4 (already 10 days without supplement); code 72: box 1 stopped taking 1 supplement every week except weeks 5 and 7, when he stopped taking 2; in box 2 stopped taking 3 in blister 9, then did not take anything; *code 24*: takes all the first box according to family but does not record it | *code 20*: took everything from the first box and first 5 capsules from the second box (5/56); *code 13:* Box 1/registration is not delivered, given that in the last two months he has used psychotropic drugs abusively, it was decided to remove him from the study after v4.; *code 47*: he took everything from the second box, two first blister packs and 4 from the third, then left it; *code 76*: large box, take everything until suspending after the first 2 cp of the 10th blister |
| V5 | - | code 70: take everything up to blister 13 first three, then nothing |
| V6 | *code 38*: Probably 100%, see registration adherence, 4-8 empty blisters, 1-3 missing, does not register adherence; *code 98*: does not deliver blister 16, in adherence all except 2 of blisters1; *code 41*: Probably 100%, see registration adherence, 4-8 empty blisters, 1-3 missing, does not register adherence; *code 96*: delivers 8 empty blister packs (1,8,10,11,13,14,15,16), questionnaire: take it every day; code 21: same number 53/53 both boxes and register | *code 33*: delivery box 1 with only one blister pack, empty, and box 2 takes everything up to the last two weeks per visit 6 somewhat early, empty records; *code 42*: Probably 100%, see registration adherence, 4-8 empty blisters, 1-3 missing, does not register adherence; code 17: box 1 delivered with only one empty blister, box 2 with all empty blister packs, the record confirms consumption; *code 46*: 8 blister is missing from box 1 but recorded every day and delivered in box 2, then nothing is taken from box 2; *code 97*: does not deliver blister 16, in adherence all except 2 of blisters1 |
| 7 | - |  |

**Supplementary Table 3.** Statistic significant differences in the interaction treatment x visit for quality of life parameters assessed by the SF-36 questionnaire. Lip: Lipchronic, intervention group; Pla: placebo, control group.

|  | **No distinguish by type of tumor** | | | | | | |  |
| --- | --- | --- | --- | --- | --- | --- | --- | --- |
|  |  | **Lip** |  | **Pla** |  |  |  |  |
|  | n (Lip/Pla) | a-LPs | 95% CI | a-LPs | 95% CI | Between group dif (Lip-Pla) | P value |  |
| **sf-36 vitality (0-100)** |  |  |  |  |  |  |  |  |
| vis 1 | 40/42 | 50,51 | (43,10 to 57,92) | 53,65 | (46,39 to 60,91) | -3,13 (-13,49 to 7,23) | 0,553 |  |
| vis 4 | 34/33 | 51,3 | (43,56 to 59,04) | 53,09 | (45,36 to 60,82) | -1,78 (-12,72 to 9,16) | 0,748 |  |
| *dif 4 vs, 1* |  | *0,79* | *(-5,31 to 6,88)* | *-0,56* | *(-6,74 to 5,61)* | *1,35 (-7,33 to 10,03)* | *0,761* |  |
| vis 6 | 27/28 | 55,41 | (47,23 to 63,58) | 48,13 | (40,09 to 56,16) | 7,27 (-4,19 to 18,74) | 0,213 |  |
| *dif 6 vs, 1* |  | *4,9* | *(-1,77 to 11,53)* | *-5,52* | *(-12,08 to 1,04)* | *10,40 (1,06 to 19,74)* | **0,029** |  |
| *dif 6 vs, 4* |  | *4,11* | *(-2,56 to 10,76)* | *-4,96* | *(-11,54 to 1,63)* | *9,06 (-0,31 to 18,42)* | *0,058* |  |
| P interact trat x vis |  | 0,123 |  |  |  |  |  |  |
|  |  |  |  |  |  |  |  |  |
|  | **Lung cancer** | | | | | | |  |
|  |  | **Lip** |  | **Pla** |  |  |  |  |
|  | n (Lip/Pla) | a-LPs | 95% CI | a-LPs | 95% CI | Between group dif (Lip-Pla) | P value |  |
| **sf-36 vitality (0-100)** |  |  |  |  |  |  |  |  |
| vis 1 | 9/8 | 62,25 | (51,07 to 73,42) | 74,17 | (62,34 to 86,00) | -11,51 (-27,94 to 4,92) | 0,151 |  |
| vis 4 | 8/7 | 69,31 | (57,75 to 80,86) | 74,72 | (62,46 to 86,97) | -5,28 (-22,12 to 11,55) | 0,529 |  |
| *dif 4 vs, 1* |  | *7,06* | *(-1,97 to 16,12)* | *0,55* | *(-8,48 to 10,17)* | *6,23 (-6,62 to 19,08)* | *0,342* |  |
| vis 6 | 7/6 | 69,81 | (58,25 to 81,36) | 68,01 | (55,37 to 80,66) | 1,56 (-15,60 to 18,72) | 0,837 |  |
| *dif 6 vs, 1* |  | *7,56* | *(-1,47 to 16,62)* | *-6,16* | *(-15,62 to 4,63)* | *13,08 (-0,33 to 26,47)* | ***0,056*** |  |
| *dif 6 vs, 4* |  | *0,5* | *(-7,84 to 8,84)* | *-6,71* | *(-15,88 to 3,19)* | *6,84 (-5,82 to 19,51)* | *0,29* |  |
| P interact trat x vis |  | **0,021** |  |  |  |  |  |  |
