# Supplementary material for: Randomized clinical trial in cancer patients shows immune metabolic effects exerted by formulated bioactive phenolic diterpenes with potential clinical benefits
Source: Front Immunol. 2025 Feb 17;16:1519978. doi: 10.3389/fimmu.2025.1519978 (PMC11872936; doi:10.3389/fimmu.2025.1519978)
Supplement: Supplementary Table 3 — Statistically significant differences in the interaction treatment x visit for quality-of-life parameters assessed by the SF36 questionnaire. [file Table2.docx]

**CONSORT-AI checklist of information to include when reporting a randomised trials of AI interventions. NO AI Intervention**

| Section | Item | CONSORT 2010 Item^a^ | CONSORT-AI Item | | Addressed on Page No^b^ |
| --- | --- | --- | --- | --- | --- |
| Title and Abstract | | | | | |
| **Title and Abstract** | 1a | Identification as a randomised trial in the title. | CONSORT-AI 1a,b Elaboration.  NO AI intervention | (i) Indicate that the intervention involves artificial intelligence/machine learning in the title and/or abstract and specify the type of model. NO AI intervention |  |
|  |  |  |  |  |  |
|  | 1b | Structured summary of trial design, methods, results, and conclusions (for specific guidance see CONSORT for abstracts |  | (ii) State the intended use of the AI intervention within the trial in the title and/or abstract. NO AI intervention |  |
|  |  |  |  |  |  |
| Introduction | | | | | |
| **Background and objectives** | 2a | Scientific background and explanation of rationale. | CONSORT-AI 2a (i) Extension  NO AI intervention | Explain the intended use of the AI intervention in the context of the clinical pathway, including its purpose and its intended users (e.g. healthcare professionals, patients, public). NO AI intervention |  |
|  |  |  |  |  |  |
|  | 2b | Specific objectives or hypotheses. |  |  |  |
| Methods | | | | | |
| **Trial design** | 3a | Description of trial design (such as parallel, factorial) including allocation ratio. Figure 2A |  |  |  |
|  | 3b | Important changes to methods after trial commencement (such as eligibility criteria), with reasons. |  |  |  |
| **Participants** | 4a | Eligibility criteria for participants. | CONSORT-AI 4a (i) Elaboration NO AI intervention | State the inclusion and exclusion criteria at the level of participants. |  |
|  |  |  | CONSORT-AI 4a (ii) Extension NO AI intervention | State the inclusion and exclusion criteria at the level of the input data. |  |
|  | 4b | Settings and locations where the data were collected. | CONSORT-AI 4b Extension NO AI intervention | Describe how the AI intervention was integrated into the trial setting, including any onsite or offsite requirements. |  |
| **Interventions** | 5 | The interventions for each group with sufficient details to allow replication, including how and when they were actually administered. reference 14 | CONSORT-AI 5 (i) Extension NO AI intervention | State which version of the AI algorithm was used. |  |
|  |  |  | CONSORT-AI 5 (ii) Extension NO AI intervention | Describe how the input data were acquired and selected for the AI intervention. |  |
|  |  |  | CONSORT-AI 5 (iii) Extension NO AI intervention | Describe how poor quality or unavailable input data were assessed and handled. |  |
|  |  |  | CONSORT-AI 5 (iv) Extension. NO AI intervention | Specify whether there was human-AI interaction in the handling of the input data, and what level of expertise was required of users. |  |
|  |  |  | CONSORT-AI 5 (v) Extension NO AI intervention | Specify the output of the AI intervention |  |
|  |  |  | CONSORT-AI 5 (vi) Extension NO AI intervention | Explain how the AI intervention’s outputs contributed to decision-making or other elements of clinical practice. |  |
| **Outcomes** | 6a | Completely defined pre-specified primary and secondary outcome measures, including how and when they were assessed |  |  |  |
|  | 6b | Any changes to trial outcomes after the trial commenced, with reasons. |  |  |  |
| **Sample size** | 7a | How sample size was determined. |  |  |  |
|  | 7b | When applicable, explanation of any interim analyses and stopping guidelines. |  |  |  |
| Randomisation | | | | | |
| **Sequence generation** | 8a | Method used to generate the random allocation sequence. |  |  |  |
|  | 8b | Type of randomisation; details of any restriction (such as blocking and block size) |  |  |  |
| **Allocation concealment mechanism** | 9 | Mechanism used to implement the random allocation sequence (such as sequentially numbered containers), describing any steps taken to conceal the sequence until interventions were assigned |  |  |  |
| **Implementation** | 10 | Who generated the random allocation sequence, who enrolled participants, and who assigned participants to interventions. |  |  |  |
| **Blinding** | 11a | If done, who was blinded after assignment to interventions (for example, participants, care providers, those assessing outcomes) and how. |  |  |  |
|  | 11b | If relevant, description of the similarity of interventions. Reference 14 |  |  |  |
| **Statistical methods** | 12a | Statistical methods used to compare groups for primary and secondary outcomes. |  |  |  |
|  | 12b | Methods for additional analyses, such as subgroup analyses and adjusted analyses. |  |  |  |
| Results | | | | | |
| **Participant flow** (a diagram is strongly recommended) | 13a | For each group, the numbers of participants who were randomly assigned, received intended treatment, and were analysed for the primary outcome. Figure 1A, 1B |  |  |  |
|  | 13b | For each group, losses and exclusions after randomisation, together with reasons. Supplementary Table 2 |  |  |  |
| **Recruitment** | 14a | Dates defining the periods of recruitment and follow-up. |  |  |  |
|  | 14b | Why the trial ended or was stopped. |  |  |  |
| **Baseline data** | 15 | A table showing baseline demographic and clinical characteristics for each group. Table 1 |  |  |  |
| **Numbers analysed** | 16 | For each group, number of participants (denominator) included in each analysis and whether the analysis was by original assigned groups. Figure 1A, 1B |  |  |  |
| **Outcomes and estimation** | 17a | For each primary and secondary outcome, results for each group, and the estimated effect size and its precision (such as 95% confidence interval). |  |  |  |
|  | 17b | For binary outcomes, presentation of both absolute and relative effect sizes is recommended |  |  |  |
| **Ancillary analyses** | 18 | Results of any other analyses performed, including subgroup analyses and adjusted analyses, distinguishing pre-specified from exploratory. NA |  |  |  |
| **Harms** | 19 | All important harms or unintended effects in each group (for specific guidance see CONSORT for harms). Supplementary Table2 | CONSORT-AI 19 Extension NO AI intervention | Describe results of any analysis of performance errors and how errors were identified, where applicable. If no such analysis was planned or done, explain why not. |  |
| Discussion | | | | | |
| **Limitations** | 20 | Trial limitations, addressing sources of potential bias, imprecision, and, if relevant, multiplicity of analyses. |  |  |  |
| **Generalisability** | 21 | Generalisability (external validity, applicability) of the trial findings. |  |  |  |
| **Interpretation** | 22 | Interpretation consistent with results, balancing benefits and harms, and considering other relevant evidence. |  |  |  |
| Other Information | | | | | |
| **Registration** | 23 | Registration number and name of trial registry |  |  |  |
| **Protocol** | 24 | Where the full trial protocol can be accessed, if available. |  |  |  |
| **Funding** | 25 | Sources of funding and other support (such as supply of drugs), role of funders. | CONSORT-AI 25 Extension. NO AI intervention | State whether and how the AI intervention and/or its code can be accessed, including any restrictions to access or re-use. |  |

^a^ We strongly recommend reading this statement in conjunction with the CONSORT 2010 Explanation and Elaboration for important clarifications on all the items.

^b^ Indicates page numbers to be completed by authors during protocol development.
