# Supplementary figures and images for: Randomized clinical trial in cancer patients shows immune metabolic effects exerted by formulated bioactive phenolic diterpenes with potential clinical benefits
Source: Front Immunol. 2025 Feb 17;16:1519978. doi: 10.3389/fimmu.2025.1519978 (PMC11872936; doi:10.3389/fimmu.2025.1519978)

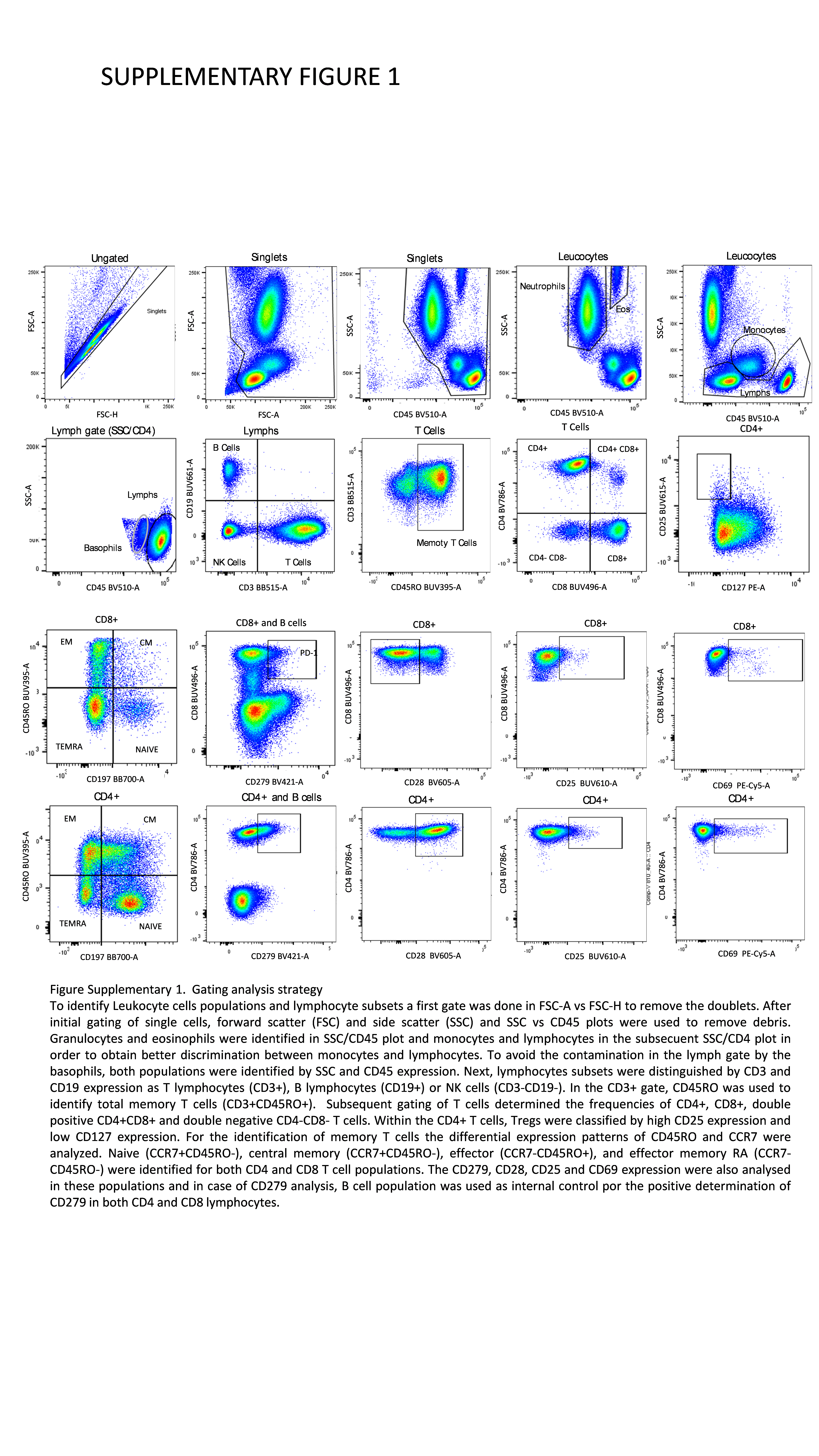

Supplement: Supplementary Figure 1 — Gating strategy for the immune phenotyping of PBMC subpopulations. [file Image1.tif]
